# Contrasting foraging strategies of seasonally segregated populations of the band-rumped storm-petrel at St Helena, South Atlantic

**DOI:** 10.1186/s40462-026-00633-1

**Published:** 2026-03-09

**Authors:** Annalea Beard, Frank Hailer, Steffen Oppel, Renata Medeiros, Leeann Henry, Alison Small, Richard A. Phillips, Robert J. Thomas

**Affiliations:** 1 Organisms and Environment Division, School of Biosciences, Sir Martin Evans Building, Cardiff University, Cardiff, Wales, UK; 2 Cardiff University-Institute of Zoology Joint Laboratory for Biocomplexity Research (CIBR), Beijing, China; 3 RSPB Centre for Conservation Science, Royal Society for the Protection of Birds, Sandy, UK; 4 School of Dentistry, Cardiff University, University Dental Hospital, Cardiff, Wales, UK; 5 Environmental Management Division, Environmental, Natural Resources & Planning Portfolio, St Helena Government, Jamestown, St Helena Island; 6 NatureSpace Partnership, 22 St. Peter’s Street, Stamford, Lincolnshire, UK; 7 British Antarctic Survey, Natural Environment Research Council, High Cross, Madingley Road, Cambridge, UK; 8 Swiss Ornithological Institute, Sempach, 6204 Switzerland

**Keywords:** Hydrobates castro, St Helena, Seabird, Foraging, Distribution, Allochrony

## Abstract

**Background:**

Allochrony can be a cause or consequence of speciation, either creating temporal reproductive isolation that reduces gene flow between diverging populations or reinforcing divergence that has already occurred through geographic isolation. The former appears to apply to band-rumped storm-petrels (*Hydrobates castro*) at some breeding sites, where there are genetically differentiated hot and cool season breeding populations. It is unclear, however, whether seasonally segregated but genetically similar populations retain the same habitat preferences or whether divergence in foraging behaviour is associated with the process of allochronic speciation.

**Methods:**

We quantified the foraging distribution of band-rumped storm-petrels at St Helena, the largest known breeding colony in the South Atlantic at which hot and cool season breeders do not appear to be genetically differentiated. Fifty-four GPS tags were deployed on experienced breeders across two hot and two cool breeding seasons. We compared foraging trip parameters, foraging effort and examined whether environmental (oceanographic and atmospheric) conditions and habitat selection varied between seasonal populations.

**Results:**

Long foraging trips lasted up to 9 days and involved travel distances of up to 3,285 km. The trip durations and distances were similar between the two seasonal populations, but directions differed markedly, resulting in pronounced differences in at-sea distributions. Adults breeding in the cool season foraged across ~ 619,000 km^2^ southeast of St Helena selecting warmer waters (~ 23.1 ± 0.7 °C). In the hot season, adults used a similarly sized area (~ 600,000 km^2^) to the southwest, but selected cooler waters (~ 21.2 ± 0.4 °C) even though overall conditions at unused but available locations were warmer (~ 23.7 ± 0.7 °C) than in the cool season (~ 20.6 ± 0.5 °C).

**Conclusions:**

Seasonal differences in oceanographic conditions likely force hot season breeders to select cool nutrient-rich waters, whereas cool season breeders may select wind or temperature conditions that minimise travel or thermoregulatory costs. This clear segregation in foraging range and habitat selection suggests that the divergence in at-sea distributions between two genetically similar seasonal breeding populations may contribute to allochrony and ultimately to sympatric speciation in the band-rumped storm-petrel at St Helena and elsewhere.

**Supplementary Information:**

The online version contains supplementary material available at 10.1186/s40462-026-00633-1.

## Introduction

The mechanisms of population differentiation and speciation are complex. A commonly invoked mechanism for speciation is that geographical barriers to dispersal limit gene flow between populations [1, 2]. One increasingly discussed alternative or complementary mechanism is termed allochronic speciation, which refers to populations that become separated by breeding time; these seasonal populations can eventually become reproductively isolated [3, 4]. In seabirds, the timing of breeding is generally determined by seasonal fluctuations in the abundance or availability of food that coincide with periods of greatest demand during reproduction [5]. However, in temperate or tropical waters that lack pronounced seasonal fluctuations, seabirds can rear chicks at different times of the year and the timing of breeding is much more variable. In extreme examples of allochrony, two seasonally-segregated populations diverge sufficiently to be classified as separate species, e.g., band-rumped storm-petrels (*Hydrobates castro*,* sensu lato*) in the Galapagos Islands [6] and the Azores Islands [7, 8]. It is unclear whether these seasonally segregated populations retain similar foraging preferences and exploit the same resources or retain preferences for the same areas at sea (albeit at different times of the year), or whether their diet, foraging behaviour and distribution have also diverged, thus offering a further barrier beyond allochrony that restricts gene flow.

The miniaturisation of reliable high-resolution global positioning system (GPS) devices has created new opportunities for tracking small pelagic seabird species, greatly improving our understanding of their foraging ecology and distribution, and the effects of a changing environment [9, 10]. Here, we use this technology to shed light on the foraging distributions of two seasonally segregated populations.

The band-rumped storm-petrel species complex includes several cryptic species, including some that are endemic to a single island group [11], as well as allochronic populations at various stages along the continuum towards full speciation. In the central South Atlantic Ocean, the band-rumped storm-petrel is strongly differentiated from conspecifics in other regions in terms of mitochondrial DNA and single nucleotide polymorphism markers from reduced representation sequencing [11]. It breeds only at two island groups, St Helena and Ascension Island. Breeders from these two island groups are only weakly genetically differentiated from each other, and there is some evidence of gene flow between them [11]. However, each group holds two seasonally segregated populations: one of these breeds in the austral summer (late September to late December, hereafter referred to as the “hot season”), and the other breeds in the austral winter (hereafter referred to as the “cool season”) [12, 13]. These two seasonal populations show limited differences in morphology and vocalisations [14] and insufficient genetic differentiation to be considered cryptic species [3, 11]. Other seabird species on both islands (e.g. red-billed tropicbirds (*Phaethon aethereus*) [15], Ascension frigatebirds (*Fregata aquila*) [16], and masked boobies (*Sula dactylatra*) [17] breed throughout the year, and storm-petrels are the only species that have segregated into mutually exclusive seasonal populations.

The overall aim of this study was to compare the at-sea spatial distribution and foraging habitat preferences of the seasonal populations of band-rumped storm-petrels at St Helena to determine whether these play a role in the extreme breeding allochrony. If foraging and habitat preferences are largely fixed, we expect that the two seasonal populations would use either geographically or oceanographically similar foraging areas, albeit at different times of the year. However, if foraging behaviour is a plastic trait, i.e., birds respond to current conditions, and those change between the two seasons, we expect that the foraging areas may differ substantially in either space or oceanographic conditions. We therefore used tracking data and remotely sensed oceanographic information to test (1) whether foraging areas were spatially segregated between the two seasonal populations; and (2) if any spatial differences in foraging areas could be explained by seasonal changes in environmental conditions and hence resource availability [18]. Our study is the first detailed examination of mechanisms that might contribute to, or maintain allochrony between seasonal populations at St Helena, contributing to our understanding of sympatric speciation.

## Methods

### Fieldwork

Fieldwork was carried out on Egg Island (15º57’57’S, 5º46’39’W), a small islet off the northwest coast of St Helena that hosts the largest known breeding population of band-rumped storm-petrels in the South Atlantic Ocean. The islet has an established array of artificial nest chambers that are used readily by both seasonal breeding populations [13].

Over three calendar years, encompassing two hot seasons (late September to late December 2017 and 2018) and two cool seasons (late March to early July 2018 and 2019), breeding band-rumped storm-petrels were equipped with small GPS devices (0.95 g, 20 × 10 × 4.5 mm; NanoFix-GEO mini, Pathtrack Ltd., Otley, UK). These were set to record a position at 120-minute intervals during incubation and 60-minute intervals during chick rearing. Three narrow strips of waterproof TESA Tape (Beiersdorf, Germany) were used to attach the GPS device to the four central rectrices. Birds were recaptured and the device removed after periods of 2–12 days. The mass of the device and attached material was 1.1 g, corresponding to 1.9–2.9% of the body mass of the tagged birds at deployment. To minimise the risk of nest-desertion, we tracked only ringed birds that had previously bred at least once. All successfully tracked birds had feather samples taken for molecular sexing (using primers 2550 F/2718R), following [19].

### Statistical analysis

All statistical analyses were carried out in the program R 3.5.1 [20]. Generalised linear mixed models (GLMMs) were implemented in the *lme4* package [21] using the ‘glmer’ function, the *t*-tests were implemented in the *stats* package [22], and the likelihood-ratio tests (LRTs) in the *lmtest* package [23]. The circular ANOVAs were generated using the ‘aov.circular’ function in the *circular* package [24]. Random forest models were implemented in the R package ‘ranger’ [25]. Significant effects in all final models were plotted using the *ggplot2* R package [26]. Values are presented as the mean ± SD unless otherwise stated.

### Assessment of the effect of tagging

To test for possible effects of the devices, we compared body mass at deployment and retrieval using a *t*-test, and breeding success between nests where an adult was tracked with an equal number of randomly selected control nests of established adult breeders each season using two logistic exposure GLMMs [27, 28]. Body mass of all breeding adults encountered during the study were also compared between hot and cool seasons using a *t*-test, to check for differences in body condition between the seasonal populations.

### Comparison of trip metrics and foraging effort

Location data from GPS loggers were downloaded using Pathtrack Archival GPS Logger software (V1.5, Pathtrack Ltd., UK). Inaccurate GPS locations were identified by visual inspection of each track, and any points requiring an unrealistic travel speed (> 50 km h^− 1^ sustained over 2 hours) were removed (< 1% of locations). Foraging trips were delineated using the ‘tripsplit’ function in the *track2KBA* R package [29]. Many Procellariiformes exhibit frequent and extended periods of egg neglect during incubation [30], which is considered a widespread adaptive response among *Hydrobates* species [31, 32]. Therefore, two trip categories were distinguished; short and long trips. Short trips were temporary excursions by adults on incubation duty who temporarily neglected the egg (hereafter referred to as ‘short neglect trips’), and these trips were determined through nest monitoring and/or GPS data from the partner. Long trips during incubation or chick rearing were foraging trips that included a series of > 5 locations where the bird travelled at least 20 km from the colony. We considered long trips to be complete if the tracked bird returned to within 30 km of the colony, even if no GPS positions were obtained upon return to the colony due to battery failure. Foraging trip parameters (total distance travelled, maximum distance from the colony and trip duration) were calculated using the ‘tripdistance’ function in the *trip R* package [33, 34]. The total distance travelled was calculated as the sum of all distances between consecutive GPS locations, assuming straight-line travel. To quantify the general direction of foraging, we calculated the great circle bearing from the colony to the most distal point during each trip using the ‘bearing’ function in the R package *geosphere* [35].

We used first passage time (FPT) (the time taken to cross a circle of a given radius; [36]) to identify regions of area restricted search (ARS) during long foraging trips. Each trip was converted to a trajectory, interpolated and discretized to hourly locations, and FPT analyses were implemented using the ‘fpt’ function in the *adehabitatLT* R package [37]. Individual ARS values were calculated, and the peak in the variance of log values for each independent trip was used to define a mean search scale for the population. Original locations where the FPT value was higher than the mean FPT value across all trips (i.e., > 1.0) were assumed to be foraging rather than travelling.

Bearings to the distal point of each trip were compared between hot season and cool season populations using circular ANOVAs [38]. Differences in other foraging trip parameters were examined using GLMMs with a gamma error structure and log link function. Individual identity (ID) and year were included as a cross-classified random effect to account for nonindependence of multiple trips by the same individual and any variation in foraging distances between years. The models also included sex and breeding stage as fixed effects to account for these known sources of variation [39]. For each foraging trip parameter, we constructed two GLMMs and compared these models using a Likelihood Ratio Test (LRT). One model included season (cool versus hot) as our main categorical variable of interest, as well as sex and breeding stage as fixed effects, and the corresponding null model included only the fixed effects (sex and breeding stage), excluding season. Model assumptions were checked using standard model validation approaches [40].

To investigate whether the proportion of time spent foraging differed between seasons, we calculated the proportion of time spent foraging per trip from the binary response (foraging: true/false) identified by the FPT analysis.

### Comparison of spatial distribution

To assess whether the size of the areas exploited on foraging trips differed between seasons, we calculated kernel utilisation distributions (UDs) using the *adehabitatHR* package [41]. Specifying a 1 × 1 km grid resolution, the “kernelUD” function was used to calculate the 95%, 75% and 50% UDs from all locations on long trips. The kernel smoothing parameter was chosen using the ad hoc method [42] and set to 44 km. Overlap in UDs between the two seasons, and between years for each season were quantified using Bhattacharyya’s affinity index (BA), calculated with the “kerneloverlap” function. This is a nondirectional statistical measure of affinity that ranges from 0 (no spatial similarity between distributions) to 1 (identical spatial distributions), and has been used in previous studies of seabirds [43, 44].

### Comparison of environmental conditions and habitat selection

Differences in spatial distributions between the two seasonal populations could indicate a spatial shift in the same preferred habitat or a difference in habitat preferences (use in relation to availability). We therefore conducted two complementary analyses to understand whether storm-petrels foraged in similar environmental conditions in each season. First, we directly compared the environmental conditions at used foraging locations between the two seasons to test whether birds targeted equivalent conditions at different times of the year, even though the distribution of conditions and resources around the breeding colony may change.

Second, within each season we compared the environmental conditions at used foraging locations to those at unused locations that would have been available to storm-petrels in that season, to test whether the selection preferences were similar between seasons even if the underlying conditions were not. To specify unused locations that could have been ‘available’, we used the foraging locations of birds from the opposite season (which indicates that storm-petrels are physically able to travel to those locations) and assigned those spatial locations random dates and times drawn from the temporal distribution of foraging locations of the focal season. This resulted in a set of times and spatial locations that would have been available but were not used by tracked storm-petrels in a given season. We present the distribution of these data for each environmental variable in terms of the mean and standard deviation for both groups (hot vs. cool season, and used vs. unused in each season), the mean difference and the upper and lower 95% confidence interval of the mean difference from 10,000 bootstrap random samples of each environmental variable per category (hot, cold, used and unused) [45].

To quantify the environmental conditions, we selected nine variables that may influence either the availability of prey or the distribution of storm-petrels directly [31–33]. We included atmospheric conditions such as cloud cover, air temperature and wind that may affect flight [46], and oceanographic variables such as sea surface temperature and chlorophyll a concentration that may affect prey availability [47, 48] (Table 2, S2). All variables corresponding spatially and temporally with foraging locations used in a given season, or the unused but available locations, were downloaded via the Env-Data system in Movebank [49] (Supplementary Information; Table S2). Wind speed (m/s) and direction (degrees) were computed at each location from surface wind vectors (zonal and meridional velocities, daily values at 0.75 degree resolution, obtained from the European Centre for Medium-Range Weather Forecasts (ECMWF); https://www.ecmwf.int/).

To test whether used and unused but available locations within each season, and the foraging locations between the two seasons could be distinguished based on all environmental variables simultaneously, we used a multivariate random forest algorithm. A random forest is a machine learning algorithm based on ensembles of regression trees that can accommodate many predictor variables and yields highly accurate predictions [25, 50–55], while accounting for complex interactions. We therefore expected that the random forest model would identify whether used and unused but available locations could be segregated, and if so, which variables would most effectively explain that segregation.

We fitted three random forest models; one to compare the environmental variables at foraging locations between seasons (season as response variable), and two comparing the used and unused (but available) locations in each season. The explanatory power of the models was evaluated by calculating the proportion of the response events that were classified accurately in internal cross-validation using the function ‘confusionMatrix’ in the R package ‘caret’ [56]. To test whether habitat preferences were consistent across seasons, we used the model trained with data from the cool season to predict foraging locations in the hot season and vice versa. If this approach yielded highly accurate predictions, we could infer that storm-petrels select similar environmental conditions in each season.

To quantify the relative importance of predictor variables in determining our response variables, we used a permutation procedure that calculates the loss in predictive accuracy of the random forest model after randomly permuting a given variable [53–55]. We implemented this assessment using the option ‘permutation’ in the R package *ranger* [25] and present results as relative variable importance, with the most important variable (greatest reduction in accuracy after permutation) assigned a value of 100%. We also present partial dependence plots for the most important variable to visualise the predicted response of storm-petrels to that variable in each season. Code and data to reproduce these analyses are available at: 10.5281/zenodo.18759266.

## Results

We carried out 54 GPS logger deployments on 45 individual band-rumped storm-petrels (including 13 pairs) on Egg Island. Eight individuals were tagged more than once (four twice in the same season, three individuals in consecutive years and one individual three times: twice in one season and in consecutive years). Six birds with GPS loggers were not re-encountered after deployment. Thirty-eight individuals were recaptured and their loggers were retrieved from 47 deployments (87%). At retrieval, one bird had lost all four central rectrices to which the logger had been attached, one logger had failed to record, and one bird had not left its nest during the deployment (Supplementary Information; Table S1). Three individuals lost 1–2 central rectrices upon removal of the logger and tape. Across both seasons, adult body mass did not differ significantly before and after logger deployment (mean mass at deployment: 52.13 ± 3.81 g; mean mass at retrieval: 51.64 ± 4.44 g, *t*_44_ = 0.923, *P* = 0.361, *n* = 45, mean mass difference 0.49 ± 3.55 g, representing 0.79 ± 6.86% of body mass). Breeding adults encountered during the study in the cool season were on average 2 g heavier (mean mass: 49.09 ± 4.89 g, *n* = 219) than those during the hot season (mean mass: 47.67 ± 5.26 g, *n* = 198, *t*_402.63_ = -2.8578, *P* = 0.004, *n* = 417, including birds that were not tracked). There was no significant difference in hatching success (LRT χ_4_^2^ = 0.2455, *P* = 0.620), fledging success (LRT χ_4_^2^ = 1.0625, *P* = 0.303) or breeding success (LRT χ_4_^2^ = 0.3551, *P* = 0.551) between nests where loggers were deployed and control nests (Supplementary Information; Figure S1).

### Comparison of trip metrics and foraging effort

Seventy-five foraging trips were identified from 38 birds tracked (20 female, 18 male), including 52 long trips, and 23 short neglect trips by 14 individuals (Table 1). Departures from the colony occurred at almost all hours (Supplementary Information; Figure S2, mean 0:39 h GMT), but most adults returned around dusk (mean 20:05 h GMT). Short neglect trips occurred more frequently during the cool season, and none extended further than 87.3 km from the colony (Table 1). Typically, when short neglect trips occurred, the adult left the nest site at dawn, stayed away for most daylight hours (mean 8.39 h, range 2–18 h), and then returned at dusk. Short neglect trip direction was usually to the west (mean bearing ± SD 271 ± 71°) and did not differ between seasons (circular ANOVA *f*_1_ = < 0.001, *P* = 0.993) (Supplementary Information; Figure S3).

**Table 1:** Characteristics of 75 foraging trips by 38 band-rumped storm-petrels (*Hydrobates castro)* tracked with GPS loggers during the hot season (late September to late December) or cool season (late March to early July) at St Helena. Note that ‘short neglect trips’ refer to excursions that leave the egg unincubated for short periods

Season	Breeding stage	N birds	N trips	Trip characteristic									
				Trip duration (h)			Max distance from colony (km)			Total trip distance (km)			Direction
				Mean ± SD	Min	Max	Mean ± SD	Min	Max	Mean ± SD	Min	Max	Mean ± SD
Short neglect trips													
Hot	Incubation	5	7	7.57 ± 5.83	2.00	16.00	18.82 ± 12.15	5.51	35.04	46.84 ± 36.58	10.95	95.07	265 ± 79°
Cool	Incubation	9	16	8.75 ± 5.56	2.00	18.00	32.64 ± 21.29	9.53	87.28	77.51 ± 54.62	19.45	188.56	285 ± 49°
Long trips													
Hot	Incubation	16	20	136.00 ± 46.94	10.00	232.00	531.25 ± 255.04	25.82	873.09	1726.89 ± 727.71	58.01	3285.02	215 ± 0.78°
	Chick rearing	1	4	38.25 ± 37.36	17.00	94.00	269.05 ± 292.09	82.40	697.79	619.06 ± 623.88	237.26	1542.20	74 ± 3.77°
Cool	Incubation	17	18	117.44 ± 57.92	10.00	212.00	587.47 ± 305.72	26.65	1043.68	1831.13 ± 971.25	59.08	3227.63	131 ± 0.92°
	Chick rearing	3	10	39.50 ± 41.34	5.00	138.00	225.74 ± 243.71	43.56	740.55	615.48 ± 698.31	94.76	2242.93	76 ± 2.67°

**Fig. 1:**
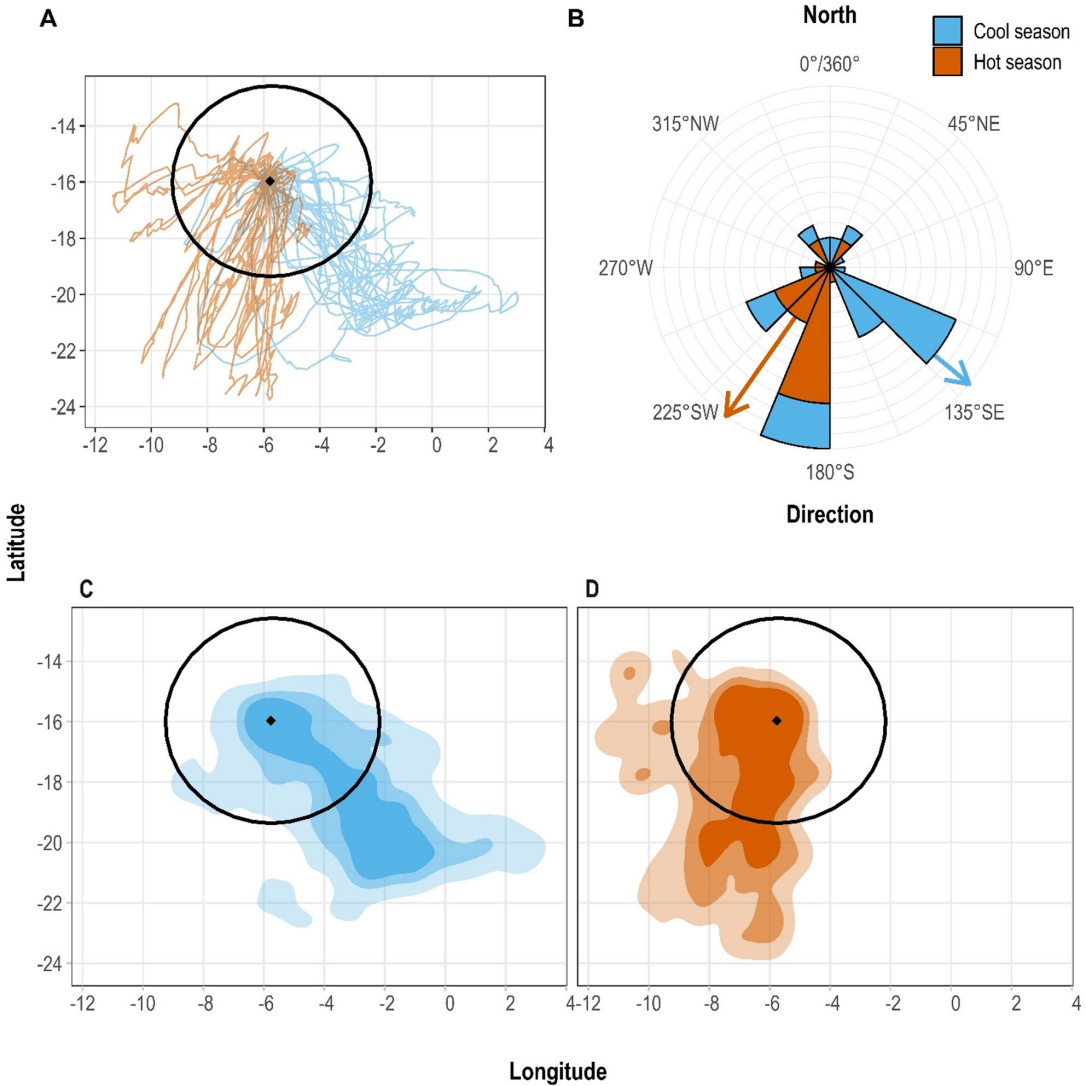
Long foraging trips of band-rumped storm-petrels (*Hydrobates castro*) (*n* = 52) tracked with GPS loggers from St Helena between 2017 and 2019 during the cool (blue) or hot (orange) breeding seasons. **A**) Individual tracks. The black diamond indicates the location of St Helena Island, and the black circle indicates a 200 nautical mile marine protected area for scale reference. **B**) Frequency of the foraging trip direction. The arrows indicate the mean trip direction per season. The height of the segments reflects the number of trips in each 22.5-degree sector. **C)** 95% (dark), 75% (medium), and 50% (light) utilisation distributions in the cool season. **D**) 95% (dark), 75% (medium), and 50% (light) utilisation distributions in the hot season

The maximum distance travelled, duration and range of foraging trips during incubation were 3,285 km, 9 days and 1,043 km, respectively (Table 1; Fig. 1A). Foraging trips were in nearly all directions when pooled across seasons (Fig. 1B), but during the incubation period, those in the cool season were usually to the southeast (mean 131 ± 68° SD, *n* = 18), and those in the hot season were usually to the southwest (215 ± 47°, *n* = 20; *F*_1_ = 27.69, *P* < 0.001, Fig. 1B). There were no differences between seasons in the mean foraging trip duration, total distance travelled or maximum distance from the colony (all *P* ≥ 0.05, Supplementary Information; Table S3). During chick rearing, trips were shorter overall, and directions did not appear to differ (Table 3), but our sample size was too small for robust inference.

### Comparison of spatial distribution

The 95% UD for long trips during the cool season was 619,000 km^2^ and centred to the southeast of St Helena (Fig. 1C). Although the 95% UD during the hot season was similar in size (600,000 km^2^), it was centred to the southwest (Fig. 1D). The two seasonal distributions had a moderate overlap, with a Bhattacharyya affinity index (BA index) of 0.502. This value was lower than the overlap in UDs between years in the same season: BA = 0.811 between the 2017 and 2018 hot season, and BA = 0.670 between the 2018 and 2019 cool season.

### Comparison of environmental conditions and habitat selection

On long trips, adults spent considerably more of their time foraging during the hot season than during the cool season (mean 49% vs. 29% of time foraging).

Based on comparisons between foraging locations that were used and those that were unused but potentially available within a given season, birds in the hot season used areas with cooler air temperatures and cooler sea surface temperatures than were unused but available at alternative locations. In contrast, birds in the cool season used warmer air and warmer waters than were unused but available at alternative locations (Tables 2 and 3). The net effect of these differences was that cool season breeders foraged in warmer waters (23.1 °C) than hot season breeders (21.3 °C), despite overall conditions at unused but available locations being cooler (20.6 °C) than in the hot season (23.7 °C, Tables 2 and 3). Tracked birds in the cool season were also more likely to forage at locations where the wind was more easterly than at alternative unused locations, whereas birds tracked in the hot season used foraging locations with a more southerly wind component than at alternative locations. There was no evidence that used and unused but available foraging locations during the cool season differed in chlorophyll *a* concentration (95% CI -0.01-0.00, Table 3), because chlorophyll *a* concentration was relatively high everywhere, but during the hot season, when concentrations were lower overall, the tracked birds selected foraging locations with higher chlorophyll *a* concentrations than at alternative locations (95% CI 0.02–0.03, Table 3).

**Fig. 2:**
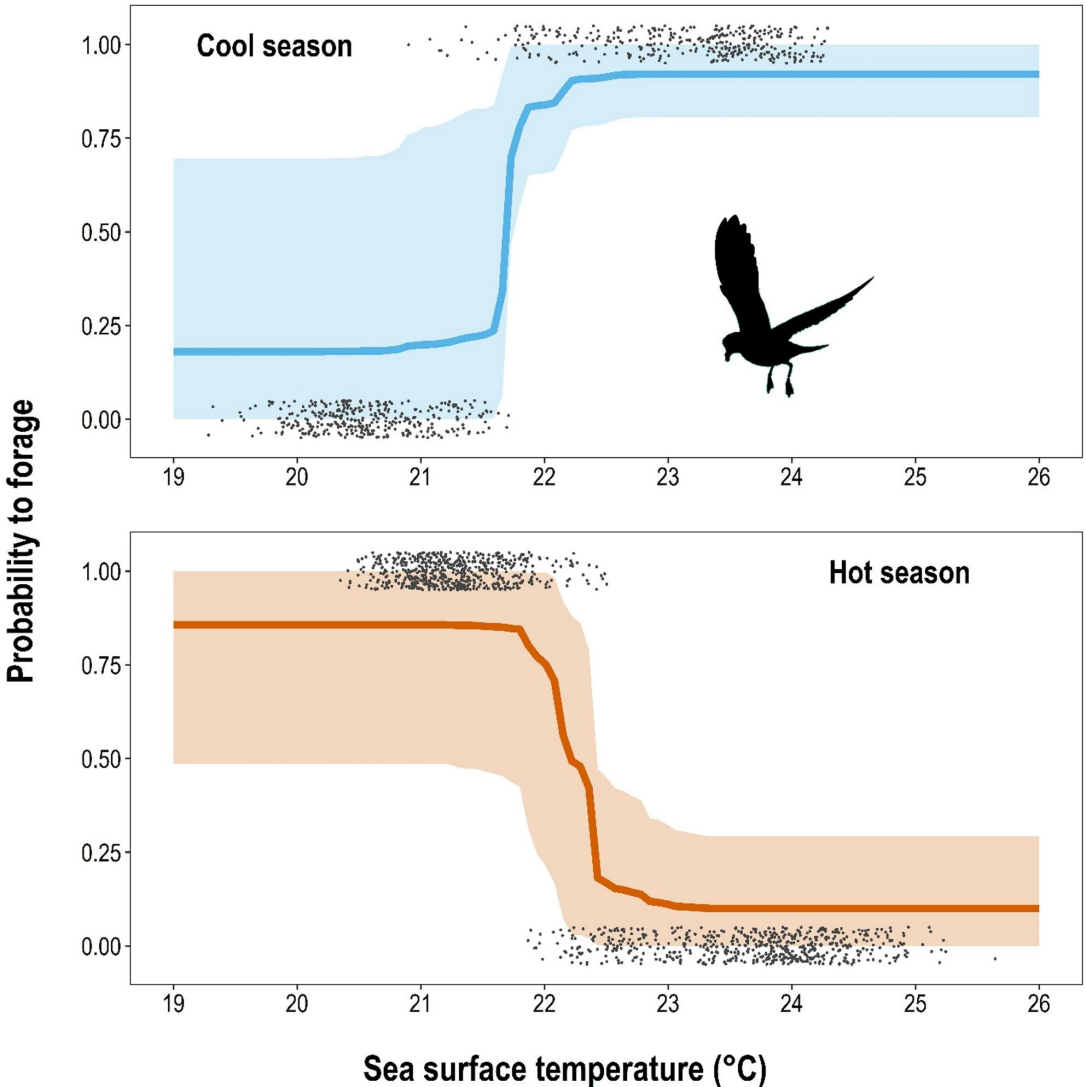
Predicted foraging response of band-rumped storm-petrels (*Hydrobates castro*) to sea surface temperature based on 52 adults tracked with GPS loggers from St Helena between 2017 and 2019 during the cool (blue, top panel) or hot (orange, bottom panel) breeding seasons. Thick line represents the median prediction of a random forest algorithm (shaded area represents 95% quantiles encompassing all variation in other environmental variables), and black dots represent raw data points of known foraging locations [1] or unused but available locations (0). Raw data are scattered vertically for better visibility

Our random forest model distinguishing foraging locations in the hot and cool season achieved 100% accuracy in cross-validation because these locations showed virtually no overlap in sea surface temperatures. Consequently, sea surface temperature was the most important variable distinguishing foraging locations in both seasons (Table 4). The two models within each season also achieved perfect segregation between used and unused but available foraging locations: the hot season model accurately predicted 100% of 1227 hot season locations, but only 4% of the 712 cool season locations. Likewise, the cool season model accurately predicted 100% of cool season locations, but only 8% of hot season locations. The most important variable in both models was sea surface temperature (Table 4). The poor model transferability between seasons was due to contrasting habitat selection, because storm-petrels selected cooler water for foraging during the hot season and warmer water during the cool season (Fig. 2).

**Table 2:** Comparison of mean values (± 1 SD) of environmental variables at locations used within a season for foraging and the mean difference (with 95% bootstrapped confidence interval) between seasons

Environmental variable	Type	Mean cool season	Mean hot season	Mean difference (lcl, ucl)
Air temperature (°C)	Atmospheric	21.68 ± 1.06	20.36 ± 0.52	-1.33 (-1.45, -1.20)
Total cloud cover (0–1)	Atmospheric	0.55 ± 0.28	0.79 ± 0.21	0.24 (0.20, 0.28)
Rain (mm)	Atmospheric	0.07 ± 0.12	0.13 ± 0.12	0.05 (0.03, 0.03)
Wind direction (º)	Atmospheric	302.10 ± 62.90	331.93 ± 10.13	29.68 (23.41, 36.90)
Wind speed (m/s)	Atmospheric	6.67 ± 2.29	7.73 ± 1.30	1.06 (0.79, 1.33)
Chlorophyll *a* concentration (mg m^-3^)	Oceanographic	0.10 ± 0.02	0.09 ± 0.04	-0.01 (-0.01, 0.00)
Sea surface temperature (°C)	Oceanographic	23.12 ± 0.73	21.27 ± 0.39	-1.85 (-1.94, -1.76)
Wave direction (º)	Oceanographic	174.19 ± 25.35	148.24 ± 23.21	-25.97 (-29.55, -22.50)
Wave height (m)	Oceanographic	2.29 ± 0.58	2.15 ± 0.39	-0.14 (-0.21, -0.06)

**Table 3:** Comparison of mean values (± 1 SD) of environmental variables at used foraging locations versus unused but available locations, and the mean difference (with 95% bootstrapped confidence interval) between used and unused locations

Environmental variable	Type	Mean used	Mean unused	Mean difference (lcl, ucl)
Cool season				
Air temperature (°C)	Atmospheric	21.68 ± 1.06	19.93 ± 0.55	1.76 (1.63, 1.88)
Total cloud cover (0-1)	Atmospheric	0.55 ± 0.28	0.66 ± 0.22	-0.11 (-0.15, -0.08)
Rain (mm)	Atmospheric	0.07 ± 0.12	0.01 ± 0.02	0.05 (0.04, 0.07)
Wind direction (º)	Atmospheric	302.10 ± 62.90	327.45 ± 9.24	-25.31 (-32.12, -19.1)
Wind speed (m/s)	Atmospheric	6.67 ± 2.29	7.84 ± 1.45	-1.17 (-1.45, -0.89)
Chlorophyll a concentration (mg m^-3^)	Oceanographic	0.10 ± 0.02	0.10 ± 0.06	-0.01 (-0.01, 0.00)
Sea surface temperature (°C)	Oceanographic	23.12 ± 0.73	20.56 ± 0.47	2.56 (2.47, 2.66)
Wave direction (º)	Oceanographic	174.19 ± 25.35	149.90 ± 9.24	24.29 (21.12, 27.44)
Wave height (m)	Oceanographic	2.29 ± 0.58	2.11 ± 0.38	0.18 (0.11, 0.25)
Hot season				
Air temperature (°C)	Atmospheric	20.36 ± 0.52	22.20 ± 0.91	-1.85 (-1.93, -1.76)
Total cloud cover (0-1)	Atmospheric	0.79 ± 0.21	0.54 ± 0.28	0.24 (0.22, 0.27)
Rain (mm)	Atmospheric	0.13 ± 0.12	0.02 ± 0.06	0.11 (0.10, 0.12)
Wind direction (º)	Atmospheric	331.93 ± 10.13	308.69 ± 68.29	23.2 (17.97, 28.76)
Wind speed (m/s)	Atmospheric	7.73 ± 1.30	6.06 ± 2.47	1.68 (1.45, 1.89)
Chlorophyll a concentration (mg m^-3^)	Oceanographic	0.09 ± 0.04	0.07 ± 0.02	0.02 (0.02, 0.03)
Sea surface temperature (°C)	Oceanographic	21.27 ± 0.39	23.65 ± 0.74	-2.38 (-2.45, -2.32)
Wave direction (º)	Oceanographic	148.24 ± 23.21	168.77 ± 29.98	-20.52 (-23.52, -17.57)
Wave height (m)	Oceanographic	2.15 ± 0.39	2.11 ± 0.60	0.05 (-0.01, 0.10)

**Table 4:** Relative variable importance in three random forest models explaining differences in environmental conditions at foraging locations of storm-petrels around St Helena. The three models explored (i) differences between seasons, or the differences between used and unused but available foraging locations within (ii) the hot and (iii) the cool season, respectively. Note that variable importance is derived from a permutation procedure and scaled to 100% for each model

Environmental variable	Between	Hot	Cool	
Sea surface temperature (°C)	100.0	100.0	100.0	
Air temperature (°C)	21.4	18.6	18.8	
Wave direction (º)	18.3	6.5	9.0	
Wind direction (º)	14.9	3.0	4.6	
Wind speed (m/s)	5.4	8.3	3.1	
Chlorophyll a concentration (mg m^-3^)	3.4	2.7	1.1	
Total cloud cover (0–1)	3.2	0.7	0.4	
Wave height (m)	3.1	1.4	0.9	
Rain (mm)	1.6	2.6	2.4	

## Discussion

We show that the seasonal breeding populations of the band-rumped storm-petrel at St Helena exhibit consistent differences in the direction of foraging trips, spatial distribution and habitat preferences. This divergence in seasonal behaviour may have contributed to sympatric speciation in this species complex [57]. 

The tracked band-rumped storm-petrels at St Helena directed their foraging trips in each season with respect to sea surface temperature and selected relatively cool areas in the hot season and relatively warm areas during the cool season. The band-rumped storm-petrels exhibited a highly pelagic foraging strategy, travelling up to 3,285 km during incubation and 2,243 km during chick rearing, which are among the longest trips recorded for any breeding *Hydrobates* species [58–63].

Although trips by storm-petrels from the two seasonal populations were similar in terms of duration, maximum distance from the colony, and total travel distance, they differed in direction and there was limited overlap in at-sea distributions. Moreover, each population was faithful to its foraging area between years, which is typical of pelagic seabirds [64]. The birds breeding in different seasons therefore appear to have diverged in terms of their habitat preferences [61] to exploit the most profitable areas in each season, which also resulted in seasonal differences in wind direction. During the hot season when marine primary productivity was lower (Table 3), band-rumped storm-petrels may have selected foraging areas to the southwest of the island to select cooler waters with higher productivity. Conversely, during the cool season the higher overall productivity of the marine environment may have given storm-petrels the opportunity to select foraging areas that optimised either travel or thermoregulation costs. Procellariiform seabirds frequently orient with crosswinds to maximise flight speeds [47, 65], but further study is needed to understand whether storm-petrels during the cool season direct foraging trips according to wind direction or for other benefits.

A higher abundance or quality of prey during the cool season [66] is supported by our finding that adults spent a lower proportion of their time foraging during the cool season than the hot, yet were on average 2 g heavier. This suggests that storm-petrels during the cool season were able to maintain body condition with less effort. However, despite the supposedly higher availability of prey, and the smaller population size during the cool season [14] they used a larger foraging area. Larger population sizes typically lead to greater competition for food resources and larger foraging ranges [67–69]. The larger foraging area of storm-petrels during the cool season despite a smaller breeding population could be caused by competition with non-breeders at sea. Very little is known about the distribution of storm-petrels outside their breeding season and birds breeding in the cool season may still interact or compete with other storm-petrels at sea that nest at a different time of the year. In addition, other life-history stages (juveniles, immatures and deferring breeders) can represent up to 81% of individuals in long-lived seabird populations [70], and these may also compete with breeders for prey. Other storm-petrel populations have larger non-breeding than breeding ranges [61, 71], but we currently have no information on the distribution of adult band-rumped storm-petrels from either the hot or cold season population at St Helena in the nonbreeding season. If they use the same foraging areas identified here for breeding birds, then the differences in space use between the allochronic populations may be to reduce intraspecific competition [72–74]. Besides competition, finer-scale processes such as prey patchiness may affect prey availability and the foraging range exploited in each season [75]. Future research using higher resolution oceanographic data, detailed prey availability assessments and tracking of birds outside the breeding seasons may offer more insights into the underlying causes of the differences in the spatial distribution of storm-petrels.

The differences in the at-sea distributions between seasonal populations may reflect inherited habitat preferences that have led to the seasonal segregation (cause), or the seasonal populations may have evolved preferences for marginally different environmental conditions that provide optimal foraging conditions in each season as a result of allochrony (consequence). Together with the temporal segregation of the two populations [13], the spatial differences in foraging distribution may eventually lead to sympatric speciation through allochrony [3, 76]. However, this segregation process appears to be at an early stage of divergence given the limited genetic differentiation between the seasonal populations on St Helena [11]. As storm-petrels can live for more than 30 years [77], genetic drift is slow, and genetic divergence is expected to lag behind ecological differentiation [78]. Hence, the segregation in foraging ranges between seasons could represent changes that are too recent or too temporally unstable to have left strong signals of population genetic structure. In contrast, Townsend’s and Ainley’s storm-petrels (*H. socorroensis* and *H. cheimomnestes*) are sufficiently distinct genetically that they are considered separate species, yet show limited spatial overlap during the nonbreeding period [79]. Our study only tracked breeding birds during the breeding season, and the spatial distribution of birds outside the breeding season is unknown. Future comparisons of nonbreeding distributions of the seasonal populations of band-rumped storm-petrel in the Atlantic may offer further insights.

## Conclusions

We show that seasonal populations of band-rumped storm-petrels breeding at St Helena use foraging areas in different directions from the colony and that this seems to result from trade-offs that may relate to seasonally varying abundance or distribution of prey. Further work is needed to understand the mechanisms leading to this seasonal divergence, and whether these spatial differences contribute to the behavioural isolation that may eventually result in allochronic speciation.

## Supplementary Information

Below is the link to the electronic supplementary material.

Supplementary Material 1

## Data Availability

The GPS dataset is available in the BirdLife Seabird Tracking Database and Movebank (ID 964155864), code is available at: 10.5281/zenodo.18759266.
