# Supplementary material for: Contrasting foraging strategies of seasonally segregated populations of the band-rumped storm-petrel at St Helena, South Atlantic
Source: Mov Ecol. 2026 Mar 9;14:18. doi: 10.1186/s40462-026-00633-1 (PMC13003698; doi:10.1186/s40462-026-00633-1)
Supplement: Supplementary file 1 — Supplementary Material 1 [file 40462_2026_633_MOESM1_ESM.docx]

# Movement Ecology

# Research article entitled “Contrasting foraging strategies of seasonally segregated populations of the band-rumped storm-petrel at St Helena, South Atlantic”

# Supplementary Figures and Tables


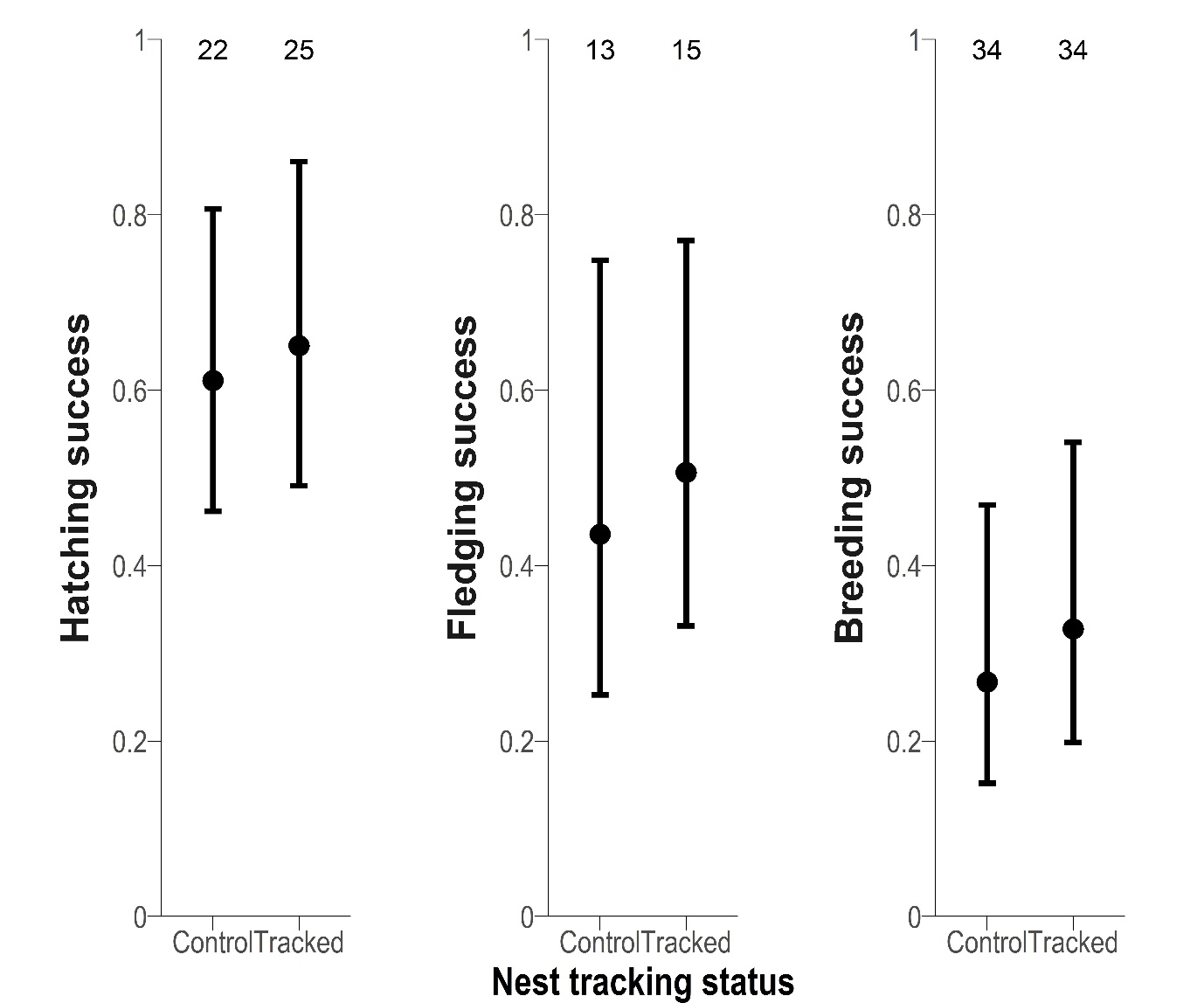


**Figure S1.** Comparison of hatching, fledging and breeding success of band-rumped storm-petrel (*Hydrobates castro*) at St Helena at 34 control nests and 34 nests where an adult was tracked in 2017-2019. The points are mean values, and the whiskers represent the 95% confidence intervals.


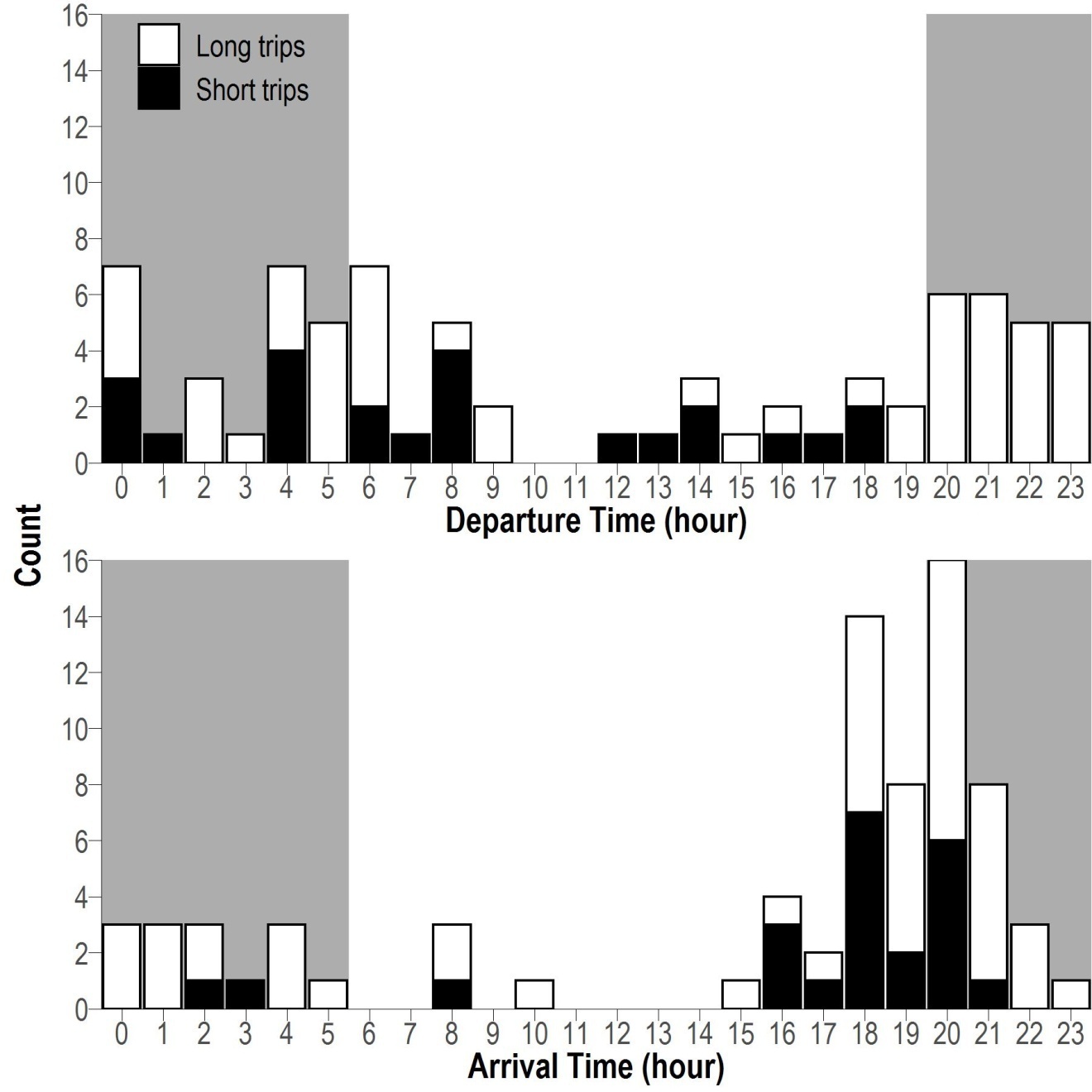


**Figure S2.** Frequency of departures (top panel) and arrivals (bottom panel) of band-rumped storm-petrel (*Hydrobates castro*) at St Helena determined from GPS tracking data. The grey shaded areas indicate the night based on mean sunset and sunrise times.


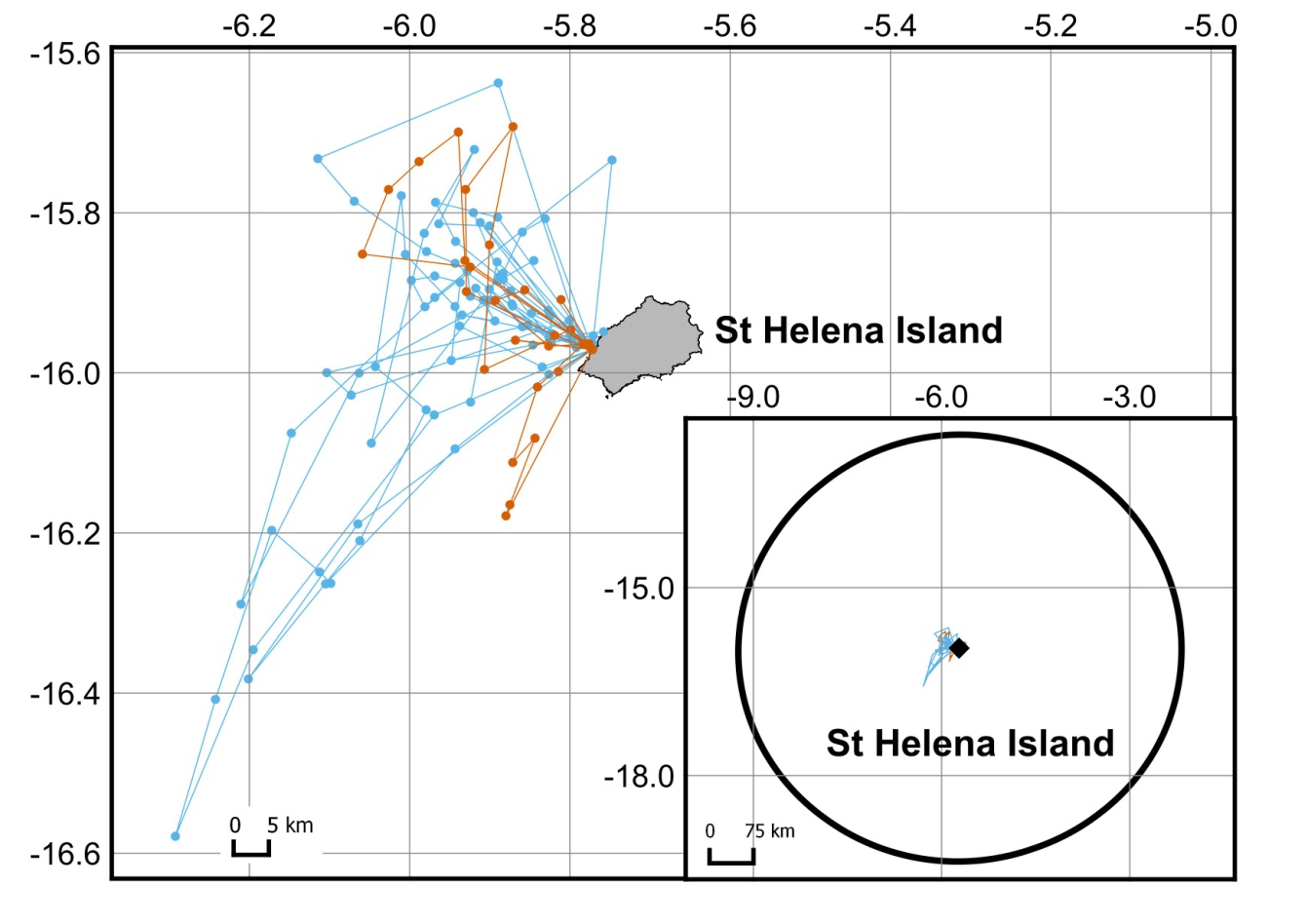


**Figure S3.** Short neglect trips made by incubating band-rumped storm-petrel (*Hydrobates castro*) during the cool (blue) and hot (orange) seasons at St Helena; note that these incubating birds neglected their egg whilst making those trips. The insert shows these short neglect trips in relation to the 200 nautical mile marine protected area (black circle) around St Helena (black diamond).

**Table S1.** Deployment details for band-rumped storm-petrels (*Hydrobates castro*) tracked at St Helena in 2017-2019.

| **Breeding stage at deployment** | **Year** | **Season** | **Number deployed** | **Temporal resolution** | **Number retrieved (%)** | **Number of trips obtained** |
| --- | --- | --- | --- | --- | --- | --- |
| Incubation | 2017 | Hot | 12 | 3 h (2); 2 h (9) | 11 (92%) | 12 L, 2 S |
|  | 2018 | Cool | 9 | 2 h | 4 (44%) | 4 L |
|  | 2018 | Hot | 10 | 4 h (1); 2 h (8) | 9* (90%) | 8 L, 5 S |
|  | 2019 | Cool | 18 | 2 h | 17** (94%) | 14 L, 16 S |
|  |  |  |  |  |  |  |
| **Total** |  |  | **49** |  | **41 (83.67%)** | **38 L, 23 S** |
| Chick rearing | 2017 | Hot | 1 | 1 h | 1 (100%) | 4 L |
|  | 2018 | Cool | 2 | 1 h | 1 (50%) | 4 L |
|  | 2018 | Hot | - | - | - |  |
|  | 2019 | Cool | 2 | 1 h | 1 (50%) | 6 L |
| **Total** |  |  | **5** |  | **3 (60%)** | **14 L** |
| **Grand total** |  |  | **54** |  | **47 (87%)** | **52 L, 23 S** |

L = long trips, S = short neglect trips, * all birds with deployed loggers recovered but one logger lost at sea, ** one logger recovered with no usable data, one incubating adult on deployment known to have hatched while the logger was active from nest checks, identified trips split between breeding stages.

**Table S2.** Sources of environmental variables included in this study.

| **Variable name** | **Spatial res** | **Temporal res** | **Source** |
| --- | --- | --- | --- |
| Air temperature (2 m above water surface) | 0.75 deg | 6-hourly | Copernicus Climate Change Service, Climate Data Store, (2023): ERA5 hourly data on single levels from 1940 to present. Copernicus Climate Change Service (C3S) Climate Data Store (CDS). DOI: [10.24381/cds.adbb2d47](https://doi.org/10.24381/cds.adbb2d47) |
| Cloud cover | 0.75 deg | 6-hourly | Copernicus Climate Change Service, Climate Data Store, (2023): ERA5 hourly data on single levels from 1940 to present. Copernicus Climate Change Service (C3S) Climate Data Store (CDS). DOI: [10.24381/cds.adbb2d47](https://doi.org/10.24381/cds.adbb2d47) |
| Precipitation | 0.75 deg | 3-hourly | Copernicus Climate Change Service, Climate Data Store, (2023): ERA5 hourly data on single levels from 1940 to present. Copernicus Climate Change Service (C3S) Climate Data Store (CDS). DOI: [10.24381/cds.adbb2d47](https://doi.org/10.24381/cds.adbb2d47) |
| Wind velocity (E-W) | 0.75 deg | 6-hourly | Copernicus Climate Change Service, Climate Data Store, (2023): ERA5 hourly data on single levels from 1940 to present. Copernicus Climate Change Service (C3S) Climate Data Store (CDS). DOI: [10.24381/cds.adbb2d47](https://doi.org/10.24381/cds.adbb2d47) |
| Wind velocity (N-S) | 0.75 deg | 6-hourly | Copernicus Climate Change Service, Climate Data Store, (2023): ERA5 hourly data on single levels from 1940 to present. Copernicus Climate Change Service (C3S) Climate Data Store (CDS). DOI: [10.24381/cds.adbb2d47](https://doi.org/10.24381/cds.adbb2d47) |
| Chlorophyll *a* concentration | 4.64 km | monthly | https://oceandata.sci.gsfc.nasa.gov/opendap/MODISA/L4SMI/contents.html |
| Sea surface temperature | 0.75 deg | daily | Copernicus Climate Change Service, Climate Data Store, (2023): ERA5 hourly data on single levels from 1940 to present. Copernicus Climate Change Service (C3S) Climate Data Store (CDS). DOI: [10.24381/cds.adbb2d47](https://doi.org/10.24381/cds.adbb2d47) |
| Wave height | 0.75 deg | 6-hourly | Copernicus Climate Change Service, Climate Data Store, (2023): ERA5 hourly data on single levels from 1940 to present. Copernicus Climate Change Service (C3S) Climate Data Store (CDS). DOI: 10.24381/cds.adbb2d47 |
| Wave direction | 0.75 deg | 6-hourly | Copernicus Climate Change Service, Climate Data Store, (2023): ERA5 hourly data on single levels from 1940 to present. Copernicus Climate Change Service (C3S) Climate Data Store (CDS). DOI: [10.24381/cds.adbb2d47](https://doi.org/10.24381/cds.adbb2d47) |

**Table S3.** Summary of generalised linear mixed models (GLMMs) and likelihood ratio tests (LRT) on the effects of season on trip characteristics of band-rumped storm-petrels (*Hydrobates castro*) at St Helena, South Atlantic tracked with GPS loggers. The marginal and conditional R^2^ values on each model were calculated following Nakagawa & Schielzeth (2013)*.

| **Trip Parameter** | **Estimate** | ***se*** | **t** | ***P*** | **Marginal/Conditional R^2^** | **LRT** | ***LRT P*** |
| --- | --- | --- | --- | --- | --- | --- | --- |
| Total distance travelled | -0.04593 | 0.21438 | -0.214 | 0.830 | 0.337/0.337 | χ^2^ _1_ =0.046 | 0.830 |
| Maximum distance | -0.033 | 0.213 | -0.156 | 0.876 | 0.245/0.245 | χ^2^ _1_=0.024 | 0.876 |
| Duration | 0.10186 | 0.18997 | 0.536 | 0.592 | 0.425/0.425 | χ^2^ _1_=10.287 | 0.592 |

* Nakagawa S, Schielzeth H. A general and simple method for obtaining R2 from generalized linear mixed-effects models. Methods Ecol Evol. 2013 Feb 1;4(2):133–42. Doi: 10.1111/j.2041-210x.2012.00261.
